# The Emerging Role of Water Loss in Dog Aging

**DOI:** 10.3390/cells14070545

**Published:** 2025-04-04

**Authors:** Gabriella Guelfi, Camilla Capaccia, Vicente Francisco Ratto, Antonello Bufalari, Leonardo Leonardi, Luca Mechelli, Simone Cenci, Margherita Maranesi

**Affiliations:** 1 Department of Veterinary Medicine, University of Perugia, 06132 Perugia, Italy; camilla.capaccia@dottorandi.unipg.it (C.C.); vicentefrancisco.rattovalderrama@dottorandi.unipg.it (V.F.R.); antonello.bufalari@unipg.it (A.B.); leonardo.leonardi@unipg.it (L.L.); luca.mechelli@unipg.it (L.M.); margherita.maranesi@unipg.it (M.M.); 2 Istituti di Ricovero e Cura a Carattere Scientifico (IRCCS) Ospedale San Raffaele, Vita-Salute San Raffaele, Genetics and Cell Biology Division, University, 20132 Milano, Italy; cenci.simone@hsr.it

**Keywords:** dog aging, water homeostasis, aquaporins, physiological dehydration, inflammaging

## Abstract

Aging involves progressive physiological changes, including the dysregulation of water homeostasis, essential for cellular function, neuronal signaling, and musculoskeletal integrity. This review explores the emerging role of water loss as a central and underestimated driver of functional decline in aging, with a focus on the dog, both as a clinically relevant target species and as a model for human aging. Age-related alterations in water metabolism—driven by changes in body composition, aquaporin (AQP) expression, electrolyte imbalances, reduced thirst perception, and impaired urine concentration—lead to intracellular and extracellular dehydration, exacerbating functional decline. We examine molecular mechanisms of water regulation involving AQPs and osmolytes, and describe how dehydration contributes to structural and metabolic dysfunction across key biological compartments, including the kidney, brain, bone, and skeletal muscle. Physiological dehydration, a hallmark of aging, intensifies inflammaging, accelerating tissue degeneration. In particular, we highlight how water loss impairs solvent capacity, solute transport, protein conformation, and cellular communication. Despite the known role of macronutrients in geriatric nutrition, hydration remains an often-overlooked factor in aging management. We argue for its inclusion as a fourth pillar in the nutritional approach to veterinary geriatrics, alongside protein, fat, and fiber. By investigating aging-associated water loss in dogs—species that share environments and lifestyle patterns with humans—we propose hydration-centered strategies to promote healthy aging in both veterinary and comparative medicine.

## 1. Introduction

Aging is not a pathological process but represents a series of physiological changes linked to the progression of time and manifested in all animals’ later stages of life. While aging is not inherently pathological, healthy aging is achievable in humans and animals through well-structured healthcare programs [1,2]. Defined as a “low probability of disease or disease-related disability in older individuals”, healthy dog aging mirrors principles observed in humans, despite limited research on canine aging. However, canine aging is an excellent model for studying human aging [3]. Differentiating healthy aging from pathological conditions is essential in veterinary practice, where the absence of clinically evident disease is a cornerstone of this concept [1]. Among the molecular processes influencing aging, water homeostasis plays a pivotal role. Water as a solvent, transporter, and vital structural component supports cellular integrity and function, placing it on par with macronutrients like carbohydrates, proteins, and lipids [4]. Its regulation through aquaporins (AQPs) and other membrane structures is essential for maintaining cellular hydration, osmotic balance, neuronal signaling, and musculoskeletal organization, which are fundamental for physical and cognitive health. However, with age, water metabolism is altered due to multiple factors, including changes in body composition, dysregulated AQP expression, disturbances in electrolyte balance, decreased thirst perception, and a reduced ability to concentrate urine. These lead to reduced intracellular and extracellular hydration, contributing to functional decline.

This review examines how age-related changes in water dynamics may arise from molecular alterations, such as disrupted AQP functionality and other regulatory mechanisms, contributing to physiological decline in aging organisms. A deeper understanding of the molecular changes underlying aging, particularly the role of water in cellular and neuronal health, can guide the development of strategies to reduce disease risk and support evidence-based medical management and lifestyle interventions. Integrating hydration management into comprehensive healthcare strategies is essential for promoting healthy aging and improving quality of life. To advance this understanding, we provide a focused overview of AQP structure, function, and molecular responses to dehydration. AQPs are small integral membrane proteins that facilitate the bidirectional transport of water molecules across biological membranes, enabling rapid and selective flux without ion permeability. Each AQP monomer forms a functional water pore, and four monomers assemble into a homotetramer within the membrane [5]. Structurally, AQPs share a conserved architecture composed of six transmembrane α-helices and two short helical loops (B and E) that dip into the membrane from opposite sides, forming the water-selective pore. The pore’s selectivity is governed by two highly conserved features: the Asn-Pro-Ala (NPA) motifs and the aromatic/arginine (ar/R) constriction site, which together exclude protons and other solutes, maintaining electrochemical gradients [6]. At the molecular level, water loss or dehydration triggers transcriptional and post-translational regulatory mechanisms affecting AQP expression, localization, and gating. In mammals and plants alike, osmotic stress induces the upregulation of specific AQPs to enhance water conservation or uptake [7]. The phosphorylation, ubiquitination, and trafficking of AQPs allow cells to fine-tune membrane water permeability in response to environmental conditions. For instance, the phosphorylation of AQP2 in renal epithelial cells controls its insertion into the apical membrane in response to vasopressin, a key adaptation to systemic dehydration [8]. Emerging evidence suggests that prolonged dehydration or oxidative stress can alter AQP conformation, stability, and oligomeric organization, potentially compromising water transport efficiency and leading to dysregulated cell-volume control [9].

## 2. Aging and Water Balance

*Aging dysregulates water balance, impairs kidney function, and reduces thirst sensitivity, increasing dehydration risk.*

**Figure 1:**
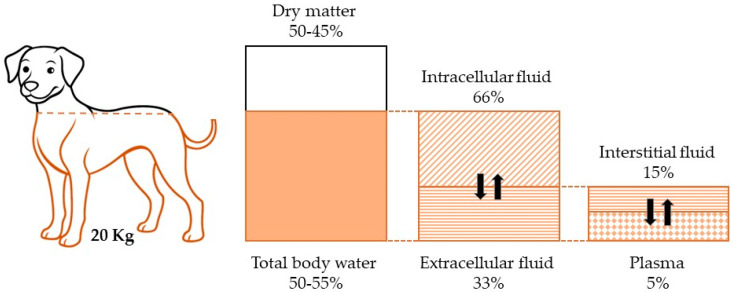
The figure illustrates the distribution of body fluids in a 20 kg dog, showing that water-based fluids account for 60% of the total body weight (highlighted with an orange border). Of this 60%, 40% is intracellular fluid, while the remaining 20% is extracellular fluid. Within the extracellular compartment, 15% consists of interstitial fluid, and 5% is plasma.

Water is an essential body nutrient because it supports various physiological functions. Water is the most abundant molecule in the body, constantly recycled and subject to strict control mechanisms to maintain an adequate level to perform all its functions [10]. The total body water (TBW) in a 20 kg dog is just over 12 L [6]. Dog TBW percentage varies with sex, nutritional status, muscularity, adiposity, and age. Puppies and young dogs have a higher water content, approximately 70–80% of body weight. As dogs age, this percentage tends to decrease, falling to 50–55% in older dogs. This decline is mainly due to muscle mass reduction and adipose tissue increase, since muscles contain about 73% water, while fat contains only 15% [11]. TBW is distributed in intracellular and extracellular compartments. Two-thirds of TBW is contained within the cells (intracellular water), serving as the primary determinant of cell volume and as the solvent for essential water-soluble cellular processes, including enzymatic reactions. The remaining one-third of TBW is located outside the cells (extracellular water). The extracellular fluid compartment can be divided into plasma, interstitial, and transcellular fluids. Plasma, the liquid component of blood, is essential for transporting nutrients and removing metabolic waste products. Transcellular fluids are liquids found in specific body spaces, such as cerebrospinal fluid (CSF) or synovial fluid, with specialized functions of protection and lubrication. Interstitial fluid fills the spaces between cells, connecting the intracellular and the intravascular compartment (Figure 1) [12].

The capillary endothelium, a selectively permeable barrier, separates the interstitial and intravascular fluid. This barrier is selectively permeable to water and small molecules but impermeable to proteins. Water distribution across capillary endothelium is regulated by the balance between filtration and absorption forces [13]. Hydrostatic pressure, generated by the heart’s pumping action, pushes water out of the blood vessels, while oncotic pressure, created by plasma proteins such as albumin, pulls water back into the intravascular space. Under homeostatic conditions, this equilibrium maintains the stability of the volume of each fluid compartment [12,14]. This balance is primarily regulated by osmolarity, defined as the total concentration of solutes, including ions, proteins, and other molecules in the fluid compartment [15]. The osmolarity differs between the intracellular and extracellular spaces and is the most important determinant of the size of each compartment [16]. The osmotic gradients drive the water movement between extracellular and intracellular compartments [15]. To maintain osmotic balance, dogs acquire water through three main sources: free available water, food, and metabolic reactions. Free drinking water makes up the majority of daily water intake. While humans can control water availability, they cannot directly increase consumption, as a dog cannot be persuaded to drink more. Water intake from food varies depending on the type of diet. Dogs fed dry food assume less than 10% moisture and therefore require a greater water intake to maintain hydration. In contrast, dogs fed wet food receive 65–75% moisture from their diet, substantially contributing to their daily water intake. Metabolic water is made during the oxidation of macronutrients such as carbohydrates, fats, and proteins and represents a minor but important contribution to total water intake [17,18]. Daily fluid requirements vary according to the size of the animal. Large animals (with a low mass/body surface-area ratio) require less fluid per kilogram on an individual basis than small animals (with a high mass/body surface-area ratio) [19]. In dogs, the normal water balance of a healthy dog is generally calculated as 50 mL per kg of body weight per day but can vary from 40 to 60 mL/kg/day. This calculation is based on the average amount a dog loses through sensible losses (measurable in urine and feces) and insensible losses (unmeasurable; mainly through breathing/sniffing) [20]. The association between physiological aging and water changes leading to a negative water balance and an increased risk of dehydration is well established and widely recognized [21]. Aging is associated with impairments in water metabolism due to multiple factors, including alterations in body composition, dysregulated AQP expression, and disturbances in electrolyte balance. Additionally, the thirst response declines with age, and the kidneys become less effective at concentrating urine. Impaired physical abilities, reduced mobility, and difficulties with eating and drinking further contribute to dehydration risk in older adults [22]. Finally, environmental factors play a significant role, as aging reduces the body’s ability to adapt to external conditions [23,24].

To replace the water lost mainly through urine, saliva, feces, and respiration, two key homeostatic mechanisms help maintain this balance: the release of arginine vasopressin (AVP) and the stimulation of thirst. When plasma osmolarity increases by 1–2%, the antidiuretic hormone AVP is synthesized by magnocellular neurons in the paraventricular and supraoptic nuclei of the hypothalamus, packaged in vesicles, and transported to the posterior pituitary where it is stored until released into the circulation. In addition, parvocellular neurons co-synthesize and release AVP into the superior pituitary artery, which supplies the anterior pituitary [25]. When arterial volume decreases by 8–10%, baroreceptors located in the pulmonary and renal arteries trigger AVP and renin release [26]. During water depletion, the AVP promotes kidney water reabsorption by increasing the collecting duct expression of AQP water channels. Specifically, AVP acts through the V2 receptor (V2R), which is located on the basolateral membrane of the renal principal cells. This binding increases cyclic adenosine monophosphate (cAMP) levels and consequently enables the activation of protein kinase A (PKA), which phosphorylates the water channel AQP2, at S256 [27]. This phosphorylation allows for the translocation of AQP2-bearing vesicles from an intracellular pool to the apical plasma membrane where AQ2 promotes the reabsorption of water from tubular fluid into the blood, allowing the kidney to excrete concentrated urine and conserve water [28]. Investigating the regulation and expression of AQP2 in aging individuals, both in humans and dogs, would be of significant interest, as no specific studies have yet explored its role in age-related physiological changes.

When urinary water reabsorption—the first defense against dehydration—is insufficient to maintain plasma osmolality, thirst is induced to increase water intake. In dogs, it has been shown that thirst occurs with a 0.5% to 1% loss of body water [29,30]. The osmoreceptors that regulate AVP secretion in the posterior pituitary also directly monitor changes in plasma osmolality, activating the mechanisms needed to generate thirst. Thus, spontaneous drinking behavior is driven by the perception of thirst to restore body water balance [31].

**Figure 2:**
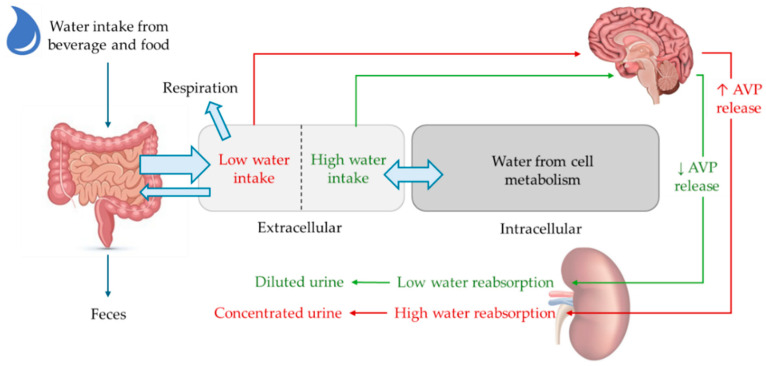
Homeostatic regulation of water balance. Water is ingested into the body and absorbed primarily in the small intestine, then enters the bloodstream and is distributed between two main compartments, intracellular (60%) and extracellular (40%). The water balance between these compartments is regulated by osmotic pressure and the concentration of electrolytes, especially sodium and potassium. The kidneys play a critical role in maintaining water balance. The antidiuretic hormone arginine vasopressin (AVP) is produced in the hypothalamus and stored in the posterior pituitary. With limited water intake, plasma osmolality increases (red arrows), and AVP is released into the bloodstream and reaches the kidneys, increasing the permeability of the collecting ducts and allowing more water to be reabsorbed into the bloodstream. As a result, urine becomes more concentrated, and less water is excreted. On the other hand, when water intake is high, plasma osmolality decreases (green arrows), leading to the inhibition of AVP release. Water reabsorption by the kidney is minimized and a large amount of dilute urine is excreted.

On the other hand, when water intake is too high, AVP levels are decreased, resulting in a lack of translocation of the AQPs to the luminal membrane of the collecting duct cells; the water permeability of the cells remains low and water reabsorption is absent. This mechanism promotes dilute urine excretion to maintain a narrow plasma concentration of osmoles (Figure 2) [24,32].

The aging process is characterized by a progressive decline in function that affects all organs, including the kidney [33]. Many aspects of renal function, such as creatinine clearance, glomerular filtration rate, and maximum urine-concentrating ability, decline with age [24]. It has been observed that basal AVP is higher in the elderly and that there is an increased excretion of AVP in response to changes in osmolarity. Therefore, the decrease in urinary concentrating ability with age has been attributed to the loss of renal receptor sensitivity to AVP (AVP resistance) [34,35]. Research involving aging rat models has shown a notable decrease in V2R expression and a diminished capacity for AVP to bind to its receptor, which stimulates the cAMP/PKA pathway [33]. Other studies indicate a decline in renal medullary AQP expression with aging [36]. The thirst response declines with age, by requiring a more pronounced increase in plasma osmolality to be triggered. The osmotic threshold for thirst activation is higher than that for AVP release, meaning that only substantial changes in plasma osmolality stimulate thirst. Compared to younger individuals, older adults consume less fluid even when experiencing a similar level of thirst, indicating a higher osmotic set point for thirst and a reduced thirst sensitivity [33].

## 3. Altered Structural Role of Water and Metabolic Efficiency in Key Biological Compartments

Water maintains the structure and function of biological systems by influencing key physiological and biochemical processes. Its unique properties help regulate osmotic balance and cell-volume adaptation, ensuring proper hydration and homeostasis in response to environmental and metabolic demands. At the cell-membrane level, water is essential for membrane stability and dynamics, preserving lipid bilayer organization and enabling critical interactions involved in cellular signaling and molecular transport. Furthermore, water facilitates the rearrangement of biological macromolecules, including proteins and DNA, influencing their folding, stability, and functional interactions. In addition to its structural functions, water serves as a universal solvent and a transport medium. However, its effectiveness diminishes with age due to decreased cellular hydration and changes in molecular mobility. Notably, the only large-cohort data available in the literature refer to humans, showing—based on a population of 545 healthy individuals aged 3 to 98 years—that total body water percentage decreases by about 15–20% in older adults compared to younger individuals [37]. This reduction contributes to age-related water loss, which affects metabolic processes, disrupts biomolecular interactions, and leads to progressive tissue dysfunction. Understanding these interconnected roles of water provides insight into its impact on cellular organization, metabolic efficiency, and aging-related structural alterations.

## 4. Osmotic Balance and Cell-Volume Adaptation

*Osmotic stress reduces cell volume, impairs DNA synthesis and proliferation, triggers apoptosis, and promotes an inflammaging response.*

Water movement between extracellular and intracellular compartments is tightly regulated and influenced by differences in osmotic pressure. Water balance regulates the tonicity of body fluids, which in turn affects the movement of water and water-soluble molecules across cell membranes. This process is essential for maintaining proper cell volume and ensuring cellular function. In cells, variations in water volume can affect vital processes such as signal transduction, hormone release, nutrient and catabolite transport, enzyme activity, and cell proliferation [38,39]. Age-related changes in water balance may also impact cell stability, as evidenced by a study showing that dog erythrocytes become more osmotically and mechanically fragile with aging, increasing their susceptibility to hemolysis and suggesting that aging negatively affects erythrocyte membrane stability [40]. To prevent these changes, mammalian cells rely on mechanisms that dynamically adjust water movement in response to variations in solute concentrations [38,39]. This regulation occurs through two main ways: the simple diffusion of water across the lipid bilayer or selective facilitated diffusion via water channels AQP proteins. Randomly moving water molecules collide with the surface of the lipid bilayer and enter the solution on the opposite side. This process is slow because the hydrophobic core of the bilayer acts as a barrier to polar molecules such as water [41]. To speed up water movement, cells use AQPs, which are found in many cell types, and facilitate passive water diffusion through selective pores in response to osmotic gradients, without requiring energy. AQP presence increases the water permeability of cellular plasma membranes 5–50-fold over lipid bilayers normally permeable to water [42]. AQPs are crucial for maintaining water balance within cells, helping to regulate cell volume, and allowing organisms to adapt to osmotic fluctuations. In humans, as in dogs, at least 13 AQPs (AQP0-AQP12) have been identified, each expressed in specific tissues and classified into three groups: classical AQPs (AQP0, 1, 2, 4, 5, 6, and 8), which are mainly responsible for transporting water [40]; aquaglyceroporins (AQP3, 7, 9 and 10), which transport water, glycerol, urea, and other metabolically significant solutes (such as carbon dioxide, oxygen, nitric oxide, ammonium and hydrogen peroxide); and intracellular AQPs (AQP6, AQP11 and AQP12), whose functions are still being studied [43]. AQPs are predominantly expressed in organs responsible for water uptake and regulation, such as the kidneys, the gastrointestinal tract, salivary glands, sweat glands, lacrimal glands, skin epithelium, and erythrocytes, which undergo continuous osmotic changes.

With aging, body water content gradually declines, increasing extracellular osmolarity and leading to a condition known as age-related hyperosmotic stress. These osmotic gradients drive water out of cells into the extracellular space to balance solute concentrations, resulting in cell shrinkage and dehydration. The consequent rise in plasma osmolarity also increases blood viscosity, which impairs circulation. As a result, neurons in the central nervous system may experience hypoxia and nutrient deficiencies, ultimately leading to cell death [44]. Mammalian cells have evolved several mechanisms to compensate for hyperosmotic stress and restore osmotic balance. These include the activation of ion transporters that allow sodium to enter the cell to balance osmolarity (although this can cause severe ionic imbalances), the synthesis of inert intracellular organic molecules such as taurine, betaine, myoinositol, and sorbitol, the upregulation of AQP gene expression to facilitate water movement, cytoskeletal rearrangement to maintain cell volume, the activation of antioxidant enzymes to counteract increased reactive oxygen species (ROS) and oxidative stress, and the activation of autophagic degradation [45]. Nevertheless, the body’s osmotic balance remains primarily regulated by AVP. In aging mammals, including dogs, age-related hyperosmotic stress disrupts various homeostatic processes within cells, impairing DNA synthesis and repair, transcription, translation, protein degradation, mitochondrial function, and intracellular signaling. These disruptions contribute to cell-cycle arrest, reduced cell proliferation, increased oxidative stress, and the activation of apoptotic pathways, all of which are associated with cellular senescence and age-related tissue degeneration [46,47].

As cellular and nuclear volumes decrease, intracellular macromolecular concentrations increase, leading to mechanical changes, including increased cell stiffness, altered protein folding, protein trafficking, chromatin condensation, and nucleocytoplasmic transport alteration [48]. Cell shrinkage also causes significant mechanical stress on the nucleus, leading to DNA strand breaks, the activation of G2 and G1 cell-cycle checkpoints, and the upregulation of DNA damage-inducible genes such as GADD45 and GADD153, ultimately resulting in cell-cycle arrest [48]. Together, the positive regulation of these proteins results in cell-cycle arrest [48]. Apoptotic processes are also initiated, including nuclear condensation, DNA fragmentation, caspase activation, the appearance of apoptotic bodies, and the translocation of phosphatidylserine to the extracellular surface of the plasma membrane. Intracellular water loss negatively affects protein structure and function, including impaired enzymatic activity. Polyubiquitinated proteins have also been observed to accumulate in cells exposed to hyperosmotic stress [48]. Hyperosmotic stress has also been associated with a strong inflammatory response due to increased synthesis and secretion of several inflammatory cytokines, including tumor necrosis factor-α, interleukin-1β, interleukin-6, interleukin-8, and interleukin-18 [48]. Several studies have demonstrated that chronic water loss and the resulting hyperosmotic stress lead to the increased production of ROS, causing oxidative stress that damages proteins, lipids, and DNA. In response, cells activate autophagy as a protective mechanism to remove damaged components and restore intracellular homeostasis. In aging organisms, the loss of water and impaired AQP function are common, making the ROS–autophagy axis especially relevant. Persistent oxidative stress contributes to inflammaging and cellular senescence, while dysregulated autophagy may exacerbate tissue degeneration. Experimental evidence shows that hyperosmotic stress increases ROS production and activates autophagy as part of the adaptive response to restore homeostasis, supporting the hypothesis that dehydration-induced osmotic stress initiates a cascade of oxidative and autophagic responses contributing to age-related decline [27,48]. The persistent pro-inflammatory state is a hallmark of inflammaging a process associated with chronic inflammatory diseases, carcinogenesis, and premature aging. Furthermore, hyperosmotic stress contributes to metabolic disorders, cardiovascular and renal diseases, and an increased risk of mortality, all of which are exacerbated by age-related inflammation [49,50].

## 5. Membrane Stability and Dynamics

*Dehydration compromises cell-membrane stability and dynamics, reducing fluidity, increasing stiffness, and impairing membrane protein function.*

Water is an important constituent of biological membranes and determines their structure and function [51]. Several experimental, computational, and theoretical studies have shown how the properties of water and ionic aqueous solutions change in the vicinity of membranes and, in turn, how the properties of membranes depend on the presence of aqueous solutions [52,53,54].

Biological membranes contain a variety of molecules, including membrane proteins, cholesterol, glycolipids, and ion channels. Their structure is provided by the presence of phospholipids that form a bilayer. The phospholipid polar-head groups are exposed to the aqueous environment forming hydration shells that stabilize the bilayer by maintaining membrane integrity and modulating interactions with proteins, ions, and other biomolecules essential for cellular functions [55]. The hydration layers surrounding biological membranes play a crucial role in regulating the activity of membrane proteins, including ion channels and receptors involved in physiological functions [56].

Water interacts with lipid-head groups through hydrogen bonding, forming two distinct hydration layers: hydration water (tightly bound) and confined water (weakly bound). The hydration water, closely associated with phosphate and carbonyl groups, maintains the surface charge and dipole potential. The confined water modulates membrane properties such as elasticity, surface charge, and protein interactions. Changes in hydration levels influence ion-channel gating and ion flux, while structured interfacial water extends into the cytoplasm, facilitating membrane–protein interactions, enzymatic activity, and signal transduction [56].

Water loss associated with aging alters membrane mechanics, increasing the likelihood of significant changes in membrane mechanical properties. This reduction in cellular water content leads to increased macromolecular crowding, which decreases protein solubility and promotes the formation of high-molecular-weight protein aggregates. These alterations contribute to the stiffening of the membrane, reduced fluidity, and impaired cellular function, ultimately playing a key role in the senescence process [57]. Furthermore, hypertonic conditions cause cell shrinkage, increasing membrane curvature and potentially creating bilayer defects [56]. Dehydration also alters the lipid composition of cell membranes and the function of membrane proteins essential for nutrient transport and signal transduction [58]. Phospholipids, major components of cell membranes, form lipid bilayers that act as barriers to protect the cell. Changes in hydration levels affect the mobility of polar headgroups, which modifies the electrical properties of the membrane, including its surface charge and dipole potential, and enhances its responsiveness to environmental variations. Dehydration can induce oxidative stress, leading to lipid peroxidation and compromising membrane integrity. These alterations in phospholipid composition can lead to increased membrane stiffness and decreased fluidity, affecting cellular processes and potentially contributing to aging [59,60].

## 6. Biological Macromolecule Rearrangement: Proteins and DNA

*Age-related dehydration induces conformational changes in DNA and proteins, compromising their structural integrity and function, and leading to cellular dysfunction and genetic instability.*

Water in mammalian cells plays a key role in the formation and stabilization of the structures of biological macromolecules. DNA, proteins, and lipids depend highly on water to maintain their proper conformation and function. Without adequate hydration, these macromolecules can lose their structural integrity, leading to serious disruptions in cellular processes. Because of its highly ionic nature, the water interacts strongly with nucleic acids [61] and plays a crucial role in maintaining the double helix structure of DNA, which depends on a delicate balance of energy contributions in an aqueous solution. These water layers are essential for maintaining the stability of the B-form structure of DNA, the biologically active conformation, requiring approximately 30% water by weight to preserve its native conformation in the crystalline state [62,63]. Water hydrates both the major and minor grooves of DNA by binding to polar atoms at the edges of base pairs. Water in the minor groove region forms a “hydration plug” cluster of relatively immobile water molecules in the minor groove of AT-rich DNA. This hydration can act as a “hydration fingerprint” for a given DNA sequence. In other words, how water molecules bind to and interact with a specific DNA sequence is distinctive and can be differentiated from other sequences. This occurs because the arrangement of nitrogenous bases and their affinity for water influence the formation of the hydration shell around the DNA, generating a specific and recognizable pattern. This allows proteins to sense the base sequence from outside the groove, enabling the rapid and unambiguous detection of the DNA sequence [64]. In an in vitro study using lysozyme as a model protein, Phan-Xuan et al. demonstrated that progressive dehydration led to measurable structural rearrangements detectable by SAXS/WAXS, indicating that protein-folding states are tightly linked to hydration levels [65]. Under aging water-loss conditions, water reduction could lead to significant conformational changes in DNA. These alterations could be mitigated through intervention strategies such as caloric restriction, improved sleep quality, increased physical activity, and targeting genes associated with longevity [66]. The best-known DNA structural change is from the B-DNA form, which is the predominant conformation under physiological conditions, to the A-DNA form. This B-to-A transition involves a change from a 10.5 to a more compact 11 base-pair-per-turn right-handed helix with altered helical parameters. Such conformational changes can disrupt normal DNA functions, including replication and transcription, leading to genetic instability [67,68].

Beyond DNA, changes in the hydration state also interfere with protein activity, as hydration forces play a crucial role in maintaining structural stability, primarily by participating in hydrogen bonding and weakening electrostatic interactions [69]. Water hydrates the peptide backbone and guides its structural assembly toward its final active structure. The polar and charged groups of proteins, including hydroxyl, amide, and carboxyl groups, primarily mediate interactions with water, stabilizing secondary structures such as α-helices and β-sheets. Non-polar atoms on the protein surface create hydrophobic interactions that enhance the local structure of nearby water molecules [70]. Water molecules also occupy cavities or pockets within proteins, stabilizing their structure by filling empty spaces and forming hydrogen bonds with backbone atoms or side chains [71]. Water molecules also often mediate protein receptor–ligand binding interactions [72,73]. Optimizing hydrogen-bonded networks among proteins, water, and ligands is essential for stabilizing interactions and enabling molecular recognition in binding proteins and enzymes [61,74]. Notably, in a large-scale in vivo study in mice, Minton et al. observed that erythrocyte aging was associated with progressive water loss, which preceded structural damage and impaired oxygen-carrying capacity—suggesting that dehydration contributes directly to age-related macromolecular instability [57]. Protein structure and function are dramatically affected by dehydration, or the loss of water from the protein matrix [65]. When proteins lose water, they lose their proper structure and ability to interact with other molecules, compromising their biological activity [75]. In addition, a human study by Lorenzo et al. linked reduced cellular hydration to decreased skeletal muscle function and frailty in older adults, highlighting that the macromolecular rearrangements driven by water loss are not only structural but also functionally impairing [49].

## 7. Aging-Related Changes in Hydration Impair the Effectiveness of Water as a Solvent and Transport Medium

*Age-related water loss alters the solvent and transport properties of water, impairing electrolyte balance, acid-base regulation, tissue metabolism, and the efficiency of physiological systems.*

Because of its physicochemical properties, water is recognized as a universal solvent for living systems, and its presence is essential for physiological processes [76]. The high dielectric constant of water helps maintain a balance in electrostatic forces between charged particles, enabling more stable and tunable interactions, such as those occurring among proteins, ions, and other biomolecules in solution [77]. Water binds ions, helping to balance and maintain cationic and anionic species in various body compartments [78]. Dehydration reduces the ability to preserve the balance of essential electrolytes such as sodium. When water concentration is too low relative to plasma sodium content, hypernatremia develops [79]. This condition is common in the geriatric population and is associated with significant morbidity and mortality in both humans and small animal veterinary patients, such as dogs. Older animals are predisposed to hypernatremia due to age-related physiological changes such as decreased thirst, decreased urine-concentrating ability, and decreased total body water [80,81,82].

Furthermore, water is essential for regulating body fluids’ pH to ensure correct physiological function. The amphoteric nature of water allows it to act as an acid and base helping to neutralize excess hydrogen (H⁺) or hydroxide (OH-) ions. Water participates in buffering systems by stabilizing the pH of blood, which in mammals is kept within the normal range of 7.35 to 7.45, and other body fluids [83]. In elderly dogs, dehydration may impair the effectiveness of these buffering systems, promoting acid-base imbalances. If the dog loses a lot of fluid, there may be a significant loss of HCO_3_-, a condition called metabolic acidosis. The reduction in HCO_3_- leads to an increase in H⁺, which lowers the blood pH. In addition, severe dehydration can reduce tissue perfusion, causing lactic acid to accumulate and further exacerbate the acidosis. If dehydration is accompanied by prolonged vomiting, the dog loses H+-rich gastric contents from the body, causing an absolute increase in HCO3-. This leads to a pH increase causing metabolic alkalosis, which can also lead to hypokalemia (low potassium levels), further exacerbating the pH imbalance [84,85].

A dog obtains water through food, drinking, or metabolic reactions. In particular, enzymatic processes, especially hydrolysis reactions of macronutrients (proteins, carbohydrates, lipids, etc.), contribute to water production and utilization within the body [86]. Dehydration significantly affects tissue metabolism, prompting the body to break down the macronutrients to generate water. It first utilizes fats, which produce the most metabolic water, then carbohydrates, and finally proteins, whose breakdown is least efficient and can further increase water loss [83]. Water is the medium for all transport systems, enabling intercellular, interstitial, and capillary exchanges. Water ensures nutrient transport to all cells and supports the efficient clearance of metabolic byproducts, making it indispensable for the proper functioning of all organs and tissues. Therefore, dehydration affects the functioning of many systems, including the cardiovascular, respiratory, gastrointestinal, reproductive, renal, hepatic, and nervous systems [10]. Water constitutes 90–92% of blood plasma, facilitating oxygen and serving as an oxygen transport medium, as well as glucose, lipids, amino acids, electrolytes, water-soluble vitamins, hormones, and growth factors in tissues [87]. Water’s solvent properties help maintain blood fluidity and efficient exchange of substances. Inadequate blood supply leads to cell death and tissue damage. In the elderly, increased plasma osmolality raises blood viscosity, reducing tissue perfusion and potentially causing hypotension or circulatory collapse [88]. By maintaining vascular volume, water ensures the proper removal of metabolic waste products, which becomes less efficient with aging. During aging, reducing water availability hinders the removal of metabolic waste products. This can lead to increased blood levels of waste products, such as urea and creatinine, produced by the breakdown of proteins, a condition known as azotemia [89]. In addition, kidney dysfunction as a result of dehydration can reduce the excretion of ammonia, causing hyperammonemia, which can affect the nervous system and potentially lead to hepatic encephalopathy [90]. Kidney disease is a prevalent condition in older pets, approximately ten percent of dogs will develop some form of kidney disease; as kidney filtering capacity decreases with age. This condition in aging pets is also closely associated with dehydration, which can exacerbate the decline in kidney function [91].

Water has a high heat capacity, playing a fundamental role in temperature regulation and absorbing and storing large amounts of heat with minimal temperature change. This property helps regulate body temperature buffering against sudden fluctuations, ensuring thermal stability in biological systems. Unlike humans, dogs have a restricted ability to sweat, limited to their paws and small areas of skin, making respiration their primary heat-dissipation mechanism. As dogs age, they become more susceptible to dehydration, which impairs thermoregulation. Studies have shown that when dogs become dehydrated, salivation is reduced by more than 90%, compromising heat dissipation and increasing the risk of heat stroke and cardiovascular failure [92,93]. In addition, the inability to effectively dissipate heat leads to reduced muscle oxygenation and increased anaerobic metabolism, resulting in lactate accumulation and further heat production [94].

## 8. Aging-Related Water Loss and Musculoskeletal Reorganization

*Age-related water loss in bone, cartilage, and synovial fluid weakens skeletal structures, accelerates osteoarthritis progression, and impairs cartilage function.*

The water content in bone—both the water bound to the mineral phase (10%) and the free water present in pores and extracellular spaces (5%)—along with the water in cartilage (70–80%) and synovial fluid (96%) play a fundamental structural role by contributing to the stability and mechanical strength of skeletal structures [95]. Water loss plays a crucial role in the aging musculoskeletal system of many organisms, including humans, impacting the function of bones and joints [96]. Water content decreasing within the bone matrix weakens skeletal robustness, increasing the risk of fractures, compromising bone flexibility and mechanical resistance, and making it more susceptible to fractures and micro-damage. This loss of water not only affects the bone but also has a profound impact on cartilage, as it contributes to the degradation of structural components such as proteoglycans and type II collagen, leading to a significant decline in its mechanical and functional properties. Among these components, proteoglycans are essential for retaining water within the extracellular matrix, providing the cartilage with viscoelastic properties that enable it to withstand compressive forces [97]. In addition to proteoglycans, type II collagen forms a fibrillar network that maintains cartilage structural integrity and tensile strength, ensuring its ability to withstand mechanical stress without compromising function. The degradation of these structural components results in a significant loss of cartilage functionality. As a result, the cartilage becomes less elastic, losing its ability to return to its original shape after being subjected to mechanical forces. This deterioration impairs the cartilage’s capacity to absorb shocks effectively, making it more prone to wear and damage over time. Consequently, the cartilage gradually thins, leading to joint dysfunction, pain, and decreased mobility. This progressive deterioration impairs cartilage function over time, leading to a decline in its ability to support joint movement effectively [98]. As continued cartilage degrades, water contributes to systemic acid-base balance by supporting buffering systems that regulate blood pH. Among these, the bone buffering system plays a key role, utilizing carbonate (Ca^2^⁺) and phosphate (HPO_4_^2−^) reserves stored in bone tissue [94,95,96]. The transfer of calcium from bone to buffer acidic conditions leads to the demineralization of the mineral matrix, reducing bone density and increasing the risk of conditions such as osteopenia and osteoarthritis (OA). Moreover, when bone buffering capacity is impaired due to dehydration-related acidosis, the body extracts calcium from bones in an attempt to restore physiological pH. This process not only weakens bone integrity but also disrupts metabolic homeostasis [99]. Extracellular acidosis further stimulates osteoclast activity, accelerating bone resorption [100]. However, the effects of acidosis on bone remodeling can be both detrimental and beneficial, depending on whether the influence is systemic or localized. As aging progresses, the gradual loss of water in cartilage accelerates degenerative processes, compromising joint function and significantly increasing the risk of OA [101,102]. Since cartilage is avascular, it relies on synovial fluid for nutrient supply and hydration. The presence of water in synovial fluid is essential for lubricating the joint and dissipating heat generated by movement, protecting tissues from potential thermal damage. A reduction in water content compromises proteoglycan function, diminishing cartilage elasticity and resilience, and increasing susceptibility to degeneration. Aging and OA further exacerbate proteoglycan loss, impairing cartilage’s ability to retain water, thus compromising joint function and predisposing it to damage [103,104,105].

With advancing age, AQPs play a key role in OA pathogenesis, being influenced by inflammatory cytokines associated with inflammaging. Alterations in AQP1 and AQP3 expression in chondrocytes impair water transport, accelerating cartilage matrix degradation and further reducing elasticity and shock absorption capacity [106,107]. Recent studies suggest a correlation between AQPs and inflammasome activation, highlighting evidence that AQP3 and AQP9 may modulate the activation of the NLRP3 inflammasome. NLRP3, predominantly expressed in macrophages, functions as a key component of the inflammasome and detects damage-associated molecular patterns, including extracellular ATP and crystalline uric acid [108,109]. A decline in water homeostasis and AQP expression may initiate a vicious cycle that amplifies inflammasome activation [110]. AQP dysfunction further impairs cellular hydration by promoting inflammation. The resulting feedback loop amplifies the detrimental effects, contributing to chronic inflammation and tissue damage over time. Age-related changes in AQP expression may exacerbate inflammatory processes, potentially accelerating tissue deterioration. In senior dogs, inflammaging is associated with a range of cellular and molecular alterations involving the persistent activation of the innate immune system, oxidative stress, cellular senescence, and changes in the gut microbiota. The continuous activation of inflammatory pathways, particularly the NLRP3 inflammasome, leads to the excessive production of pro-inflammatory cytokines such as IL-1β, IL-6, and TNF-α, which contribute to tissue deterioration. Oxidative stress, resulting from ROS accumulation, causes cellular damage and accelerates the senescence state. This phenomenon induces the senescence-associated secretory phenotype (SASP) perpetuating the inflammatory state. Additionally, alterations in the gut microbiota, accompanied by increased intestinal permeability, can further amplify systemic inflammatory responses. The consequences of inflammaging in dogs include common degenerative conditions such as OA [111,112] and sarcopenia [113,114]. In dogs, sarcopenia has been linked to chronic inflammatory processes and metabolic dysfunctions similar to those observed in humans. Recent studies highlight the role of inflammaging in dogs, where inflammasome activation contributes to muscle deterioration. Age-related intracellular water depletion compromises muscle energy metabolism and regenerative capacity, two closely interconnected phenomena. Water decline negatively impacts muscle key energy-producing processes, such as glycolysis, the Krebs cycle, and oxidative phosphorylation. This energetic inefficiency hinders the ability to sustain muscle contraction and recover from fatigue. Simultaneously, intracellular water deficiency affects the muscle’s regenerative capacity. Muscle satellite cells, the stem cells responsible for tissue repair and regeneration, require a well-hydrated environment to activate and proliferate. Intracellular dehydration reduces their efficiency, slowing down muscle regeneration and increasing the muscle’s susceptibility to damage. Furthermore, dehydration induces a state of cellular stress, which can promote apoptosis of muscle fibers, thereby accelerating the loss of muscle mass and function. The musculoskeletal age-associated pro-inflammatory environment epigenetically remodels numerous cellular pathways through epigenetic modifications such as DNA methylation, histone modifications [115], and age-dependent alterations in non-coding RNA (ncRNA) [116].

## 9. Neuronal Signaling and Water Aging-Related Loss

*Aging-induced dehydration disrupts AQP function, reducing brain hydration, impairing nerve conduction and neuronal communication, and accelerating neurodegeneration and cognitive decline.*

The abnormalities in aging water homeostasis may be early expressions of neuronal dysfunction and progressive cognitive decline [117]. In old humans, it has been shown that neurodegenerative diseases, such as dementia and Alzheimer’s, are related to hydration status [118,119,120]. Cognitive Dysfunction Syndrome (CDS) is the canine equivalent of Alzheimer’s. CDS is one of the most common cognitive abnormalities in older dogs [121,122,123]. Studies estimate the prevalence of CDS in older dogs to be 7% to 68%, with a frequency greater than 50% in dogs >15 years [124,125,126]. The regulation of water movement through AQPs and other membrane structures plays an essential role in facilitating synaptic and axonal signaling, processes fundamental to neuronal functionality. In the central (CNS) and peripheral nervous systems (PNS), AQPs are pivotal for water balance, osmotic homeostasis, and neuronal communication [127]. Aging-related reductions in intracellular and extracellular hydration, coupled with alterations in the expression and functionality of AQPs, may exacerbate the functional decline of nervous systems. This connection underscores the potential interplay between disrupted water homeostasis, altered neuronal transmission, and the progression of cognitive disorders such as CDS, emphasizing the indispensable role of hydration in mitigating cognitive decline with age [128,129].

Alterations in AQP4 expression with advancing age have been linked to inefficiencies in CSF drainage and an increased risk of toxic protein accumulation, such as β-amyloid. A reduction in AQP4 levels in astrocytes can impair brain water homeostasis, negatively affecting synaptic transmission and metabolite clearance [130]. In the canine brain, water homeostasis is maintained by regulatory processes that modulate the expression and distribution of AQP4 and AQP9, orchestrating water movements. Under baseline physiological conditions, AQP4 and AQP9 are involved in cerebral homeostasis and the regulation of central plasma osmolarity [131]. AQP9, expressed in neurons and astrocytes, facilitates water transport and small solutes such as lactate and glycerol, which is particularly important under metabolic stress. A reduction in AQP9 activity may limit the availability of energy substrates [132]. In aged dogs, decreased cerebral hydration, nutrient supply, and reduced metabolite clearance contribute to the accumulation of toxic proteins, which has been associated with cognitive decline compared to Alzheimer’s disease in humans [133,134,135]. In the PNS, several isoforms of AQPs, primarily AQP1, AQP4, and AQP9, are expressed in various cell types, including neurons, Schwann cells, and endothelial cells. Impaired AQP functionality in peripheral nerve fibers can disrupt nerve conduction, contributing to sensory loss, reduced motor function, and pain perception disorders [136]. Physiological aging involves intracellular or extracellular water reduction, which can affect chemical and electrical gradients. This decrease in water content may also impair the structure of channel proteins because hydration is essential to maintain the three-dimensional conformation required for ion channels to function properly. Channels opening and closing impairing mechanisms can modify ion transport rates and increase the risk of nerve impulse transmission disruptions. The integrity of channel proteins is essential for proper electrical and chemical signal transmission through the neurons. In dogs, measuring changes in body weight provides a simple, non-invasive method for assessing hydration levels and adjusting fluid therapy in veterinary practice [137]. A nerve cell functions as both a source and a target of signals, a characteristic that contributes to the formation of neural networks and enables complex, multifactorial communication. Among the primary modes of communication in CNS and PNS are volumetric transmission (VT) and wiring transmission (WT), both of which are strongly influenced by the body’s water balance [138]. VT, introduced by Agnati and Fuxe [138], represents a form of chemical communication that transcends the boundaries of traditional synapses, significantly expanding the potential of neural networks. The concept of VT, further developed by Borroto-Escuela et al. [139], describes an intercellular communication mechanism occurring within extracellular fluid and CSF. Unlike synaptic transmission, VT does not require direct cell-to-cell contact but relies on two principal modalities: convection and diffusion. Convection involves the transport of molecules through fluid movement driven by forces such as blood-flow pulsations, cardiac rhythm, or CSF movement. These processes enable communication between neuronal and glial cells, allowing for the release of neurotransmitters to extrasynaptic receptors, the long-distance diffusion of neuropeptides, and molecular signaling via extracellular vesicles. The volume and water content in the body plays a crucial role in regulating VT efficiency, underscoring the importance of hydration for optimal neural communication [138]. In the context of VT and diffusion, the extracellular fluid serves a dual purpose: it acts as a transport medium for molecules and assists in regulating chemical and electrical gradients. These gradients not only affect the rate of substance movement but also influence interactions with receptors and the passage of ions across cell membranes. Nicholson and Sykova [140] conceptualized the extracellular space as a gel-like structure, where the migration of chemical messengers (hormones and neurotransmitters) and ions (primarily Na+, K+, Cl−) occurs. The exchange, including the rate and quantity of ion movement, is shaped by the volume fraction, which impacts the efficiency of molecular and ionic transport in this extracellular environment [141]. During aging, water loss in tissues, including extracellular fluid and CSF, can disrupt diffusion gradients and reduce CSF fluidity, impairing the efficiency of these communication mechanisms [142]. The WT represents another fundamental mechanism of intercellular signaling. In WT, signals travel through well-defined physical connections such as synapses, axons, and gap junctions. This mode of communication allows for rapid and highly specific transmission, essential for complex neuronal functions [143,144]. In both VT and WT, reduced water availability can contribute to delays in synaptic and axonal transmission, exacerbating the decline in aging neuronal functions [145,146]. Physiological dehydration linked to human aging negatively impacts the removal of neurotransmitter catabolites in the CNS and PNS, with significant implications for neuronal function, which contributes to progressive cognitive decline. Normal brain aging differs from pathological changes in that it involves relatively mild, yet significant, structural, biochemical, and molecular modifications. These include tissue atrophy, neurotransmitter imbalances, and the gradual buildup of cellular damage [147]. Dehydration can influence various aspects of brain physiology, such as blood viscosity and cerebral perfusion. Currently, no scientific evidence directly links water loss in nervous tissue to specific alterations in synaptic function or ion-channel dynamics during aging in humans or dogs [34]. A study demonstrated that reduced water intake in older animals is associated with changes in astrocyte density and neuronal activation, impacting circuits that regulate brain water balance [148]. In aging dogs, cognitive decline and neuropathological alterations, similar to those observed in human Alzheimer’s disease, have been linked to neurodegenerative processes associated with decreased efficiency of neuronal transport systems and reduced water content in the brain [149]. As age progresses, the thirst response to hypovolemia, hypertonicity, and dehydration diminishes, and unfortunately, the endocrine systems responsible for maintaining fluid and electrolyte homeostasis become increasingly impaired.

## 10. Water-Balance Valuation in Aged Dogs Via Visual and Instrumental Approaches

*Accurate hydration assessment in aging dogs requires combining visual and instrumental methods, as age-related changes affect traditional tests.*

Accurately assessing hydration status in aging dogs requires a multimodal approach that combines visual inspection with instrumental techniques. While visual assessments are rapid and non-invasive, their reliability may be compromised by age-related changes, such as reduced skin elasticity and alterations in body composition [150]. For instance, decreased skin elasticity compromises the reliability of the skin turgor test, while circulatory alterations can influence capillary refill time (CRT), reducing its diagnostic accuracy [151]. Instrumental techniques, including transepidermal water loss (TEWL) measurement and ultrasonography, provide objective, quantitative data but may require specialized equipment and training. Integrating both approaches allows for a more precise evaluation and timely intervention. The skin turgor test involves gently lifting a fold of skin, typically between the shoulder blades or on the forehead, and observing the time it takes to return to its normal position. In well-hydrated dogs, the skin returns rapidly, whereas in dehydrated individuals, the return is delayed or the skin remains tented. Studies indicate that evaluating skin turgor on the forehead may yield more consistent results [151]. However, factors such as advanced age, weight loss, and dermatological conditions can affect test accuracy, limiting its reliability in geriatric dogs. The CRT is assessed by applying gentle pressure to the gums until blanching occurs and recording the time required for color restoration. Although prolonged CRT may indicate dehydration or circulatory impairment, studies suggest that it is not a reliable standalone marker of hydration status, as it is influenced by systemic conditions unrelated to fluid balance [151]. Furthermore, research indicates that CRT may not reliably reflect hydration status in dogs undergoing physical activity, limiting its utility as a sole diagnostic tool in these cases [151].

Among instrumental methods, TEWL measurement quantifies the rate of water evaporation through the skin, providing insights into skin barrier integrity and hydration status [152]. The EP-2 Evaporimeter, validated for use in dogs, allows for precise TEWL assessments, making it a valuable tool for monitoring hydration in aging patients [153]. Unlike visual methods, TEWL provides objective, quantitative data, enhancing diagnostic accuracy. Another instrumental technique is skin hydration (SH) measurement, which evaluates the water content in the stratum corneum. Although studies on SH in dogs are limited, this method is widely used in veterinary dermatology and may apply to hydration monitoring in aging dogs. Its established use in dermatology highlights its potential role in assessing aging SH alterations, making it a promising complementary tool in geriatric veterinary practice. The integration of advanced instrumental techniques further improves hydration assessment. High-frequency ultrasonography, a non-invasive imaging modality, measures skin thickness and detects hydration-related changes. Research indicates that following intravenous fluid administration, significant increases in skin thickness and reductions in dermal echogenicity occur across multiple body regions in dogs, highlighting its utility for hydration monitoring [154]. Another promising technique is bioelectrical impedance analysis (BIA), which estimates body fluid volumes by measuring tissue resistance and reactance to a low-intensity electrical current. Studies have validated BIA feasibility in dogs, demonstrating significant changes in bioelectrical parameters following blood donation, suggesting its potential for hydration monitoring in clinical and research settings [154].

## 11. Conclusions

Aging profoundly alters water homeostasis in dogs, impacting physiological functions essential for health and longevity. The progressive decline in water-balance regulation contributes to metabolic and systemic dysfunctions, underscoring the importance of hydration as a critical factor in aging. While much attention has been given to macronutrients in geriatric nutrition, water remains an often-overlooked component despite its fundamental role in cellular integrity, thermoregulation, and waste elimination. Emerging evidence highlights the need for hydration-centered strategies in veterinary geriatrics, integrating tailored dietary approaches, hydration monitoring, and interventions that support AQP function and renal efficiency. Further research is needed to explore the molecular underpinnings of water metabolism in aging and to develop targeted strategies to mitigate dehydration-related decline. Understanding the interplay between water balance and aging is essential for optimizing veterinary care, ultimately improving health outcomes and quality of life in aging dogs. By prioritizing hydration as a key component of geriatric management, we can enhance longevity and well-being, offering more effective, evidence-based approaches to age-related challenges.

## Data Availability

No new data were created or analyzed in this study. Data sharing is not applicable to this paper.
